# Supplementary material for: Assessment of Individual Radiosensitivity in Breast Cancer Patients Using a Combination of Biomolecular Markers
Source: Biomedicines. 2023 Apr 7;11(4):1122. doi: 10.3390/biomedicines11041122 (PMC10136353; doi:10.3390/biomedicines11041122)
Supplement: Supplementary file 1 [file biomedicines-11-01122-s001.zip › Supplementary Figure legends.pdf]

Supplementary Figure S1

Representative images of DNA repair foci in fresh PBL collected prior RT. γH2AX and 53BP1 foci are shown in green and red, correspondently. The last column shows the merged images with DNA stained with DAPI in blue.

Supplementary Figure S2

Validation of sensitivity and specificity of *in vivo* - analyzed DNA repair γH2AX and 53BP1 foci for prediction of acute adverse reactions of BC patients in RT. Receiver Operating Characteristic (ROC) curve for analysis of γH2AX /53BP1 foci before the RT representing true positive (sensitivity) and false positive (specificity) fraction in the detection of individual RS in BC patients.

Supplementary figure S3

In vivo – defined γH2AX/53BP1 foci in the PBL of BC patients. Figure shows mean number of foci in fresh PBL isolated before RT in patients with/without late healthy tissue toxicity. The mean data from nineteen patients with no reaction, twenty-six patients with hyperpigmentation and 3 patients with erythema along with 95% confidence interval are shown.

Supplementary Figure S4

Representative images of chromosomal aberrations and MN. Image of metaphase with dicentric and ring chromosome (A) and MN (B) is shown.

Supplementary figure S5

Figure shows the level of dicentric and ring chromosomes as well as total aberrations and micronuclei. Mean values from six CHT+RT patients and seven RT patients collected before the RT (1), 24 h after 4th fraction of RT (2), one month after RT (3) are shown along with standard deviations.

Supplementary Figure S6

Figure shows the percentage of cells with dicentric and ring chromosomes as well as micronuclei in the cells from patients with late toxicity and without any late toxicity. Mean values from nine patients with hyperpigmentation and four patients without side effects as collected prior RT (1), 24 h after the 4th fraction of RT (2), one month after RT (3) are shown along with standard deviations.

Supplementary Figure S7

Validation of sensitivity and specificity of *in vitro* - analyzed 53BP1 and co-localized γH2AX /53BP1 foci 30 min after 2 Gy irradiation for prediction of acute adverse reactions of BC patients in RT. Receiver Operating Characteristic (ROC) curve for analysis of γH2AX /53BP1 foci before the RT representing true positive (sensitivity) and false positive (specificity) fraction in the detection of individual RS in BC patients.

Supplementary Figure S8

In vitro assessment of apoptosis after IR. Figure shows the percentage of live, Early apoptotic (EA) and Late apoptotic/necrotic (LAN) cells 0, 24 and 48 h after the irradiation with 0 (A) and 2 Gy (B) of γ-rays as analyzed with Annexin-V/7-AAD staining using flow cytometry. Data from four RS and four matched NOR patients are shown.
