# Supplementary material for: Assessment of Individual Radiosensitivity in Breast Cancer Patients Using a Combination of Biomolecular Markers
Source: Biomedicines. 2023 Apr 7;11(4):1122. doi: 10.3390/biomedicines11041122 (PMC10136353; doi:10.3390/biomedicines11041122)
Supplement: Supplementary file 1 [file biomedicines-11-01122-s001.zip › Table S1 info about the patients.pdf]

Table S1: The features of patients such as age, tumor nodes metastasis classification (TNM), chemotherapy status (CHT), radiotherapy (RT) fractionation and dose, usage of boost during RT and characterization of acute/late side effects. Erythema (Ery), hyperpigmentation (HP)

#	Age	TNM classification	CHT	RT (dose/fraction)	Total dose	Boost (dose)	Acute side effects	Late side effects
**1**	51	pT2N1aMX	yes	2 Gy	50 Gy	16 Gy	Ery (G1)	HP
**2**	81	pT DCIS N0M0	No	2.7 Gy	40.5 Gy	No	G0	Ery
**3**	71	pT1cpN0	yes	2.2 Gy	44 Gy	No	G0	No reaction
**4**	67	pT2pN2M0	yes	2 Gy	50 Gy	No	G0	HP
**5**	67	pT1cpN0	yes	2.5 Gy	42.5 Gy	10 Gy	G0	HP
**6**	55	pT1bpN0	No	2.5 Gy	42.5 Gy	No	Ery (G1)	No reaction
**7**	70	pT1bpN0	yes	2.5 Gy	42.5 Gy	No	Ery (G1)	HP
**8**	65	pT1bpN0	No	2.7 Gy	40.5 Gy	No	Ery (G1)	No reaction
**9**	68	pT1cpN0	yes	2.5 Gy	42.5 Gy	10 Gy	Ery (G1)	No reaction
**10**	55	pT1cpN0	No	2.5 Gy	42.5 Gy	No	Ery (G1)	HP
**11**	65	pT1bpN0	Yes	2.5 Gy	42.5 Gy	No	G0	No reaction
**12**	81	pT1bpN0	Yes	2 Gy	50 Gy	No	Ery (G1)	No data
**13**	51	pT2NmiMX	Yes	2 Gy	50 Gy	No	Ery (G2) + skin desqamation	HP + telangiectasia
**14**	73	pT1cN2M1	Yes	2 Gy	50 Gy	No	G0	HP
**15**	73	pT2pNXMX	No	2.7 Gy	40.5 Gy	No	Ery (G1)	HP
**16**	51	pT1bpN0	No	2.5 Gy	42.5 Gy	No	Ery (G1)	HP
**17**	45	pT1cpN0	Yes	2.5 Gy	42.5 Gy	No	Ery (G1)	HP
**18**	53	pT1bpN0	No	2.5 Gy	42.5 Gy	No	Ery (G1)	HP
**19**	66	pT1cpN0M0	No	2.5 Gy	42.5 Gy	No	Ery (G1)	HP
**20**	66	pT1cpN0M0	No	2.5 Gy	42.5 Gy	No	Ery (G1)	No reaction
**21**	63	T4bN0	Yes	2 Gy	50 Gy	No	Ery (G2)	HP
**22**	32	cT2cN0 (1)M0	Yes	2.2 Gy	44.4 Gy	10 Gy	Ery (G1)	No reaction
**23**	53	pT1bpN0	yes	2.5 Gy	42.5 Gy	No	Ery (G1)	No reaction
**24**	47	pT2N2M0	Yes	2 Gy	50Gy	No	G0	No reaction
**25**	43	pT1cpN0	No	2.5 Gy	42.5 Gy	9 Gy	Ery (G1)	No reaction
**26**	71	pT1bpN0 M0	No	2.5 Gy	42.5 Gy	No	Ery (G1)	Ery
**27**	64	pT2pN1a	yes	2.5 Gy	42.5Gy	No	G0	HP
**28**	56	pT2pN2M0	Yes	2 Gy	50 Gy	10 Gy	Ery (G1)	Ery
**29**	71	pT1NO	No	2.67 Gy	40.05 Gy	No	Ery (G1)	No reaction
**30**	56	pT1pN0M0	No	2.5 Gy	42.5 Gy	10 Gy	Ery (G1)	No reaction
**31**	59	pT1cpN0M0	No	2.5 Gy	42.5 Gy	10 Gy	Ery (G1)	Telangiectasia

**32**	67	pT1bpN0 M0	No	2.5 Gy	42.5 Gy	No	G0	No reaction
**33**	69	pT1bpN0 M0	No	2.5 Gy	42.5 Gy	No	Ery (G1)	HP, leakage
**34**	57	pT1cpN0M 0	No	2.5 Gy	42.5 Gy	No	Ery (G1)	No reaction
**35**	61	ypT2ypN1 cypMX	yes	2 Gy	50 Gy	9 Gy	Ery (G2)	HP
**36**	38	pT1cpN1	Yes	2.2 Gy	44 Gy	No	Ery (G1)	No reaction
**37**	56	pT1cpN1M 0	Yes	2.5 Gy	42.5 Gy	No	Ery (G1)	HP
**38**	60	pT2pN0M 0	No	2.5 Gy	42.5 Gy	10 Gy	Ery (G1)	No reaction
**39**	46	pT2pN0M 0	Yes	2 Gy	50 Gy	No	Ery (G1)	No reaction
**40**	68	pT1cpN0M 0	yes	2.2 Gy	44 Gy	No	G0	No reaction
**41**	53	pT2N0M0	Yes	2.5 Gy	42.5 Gy	10 Gy	Ery (G1)	HP
**42**	50	ypT1apN0	yes	2.5 Gy	42.5 Gy	10 Gy	Dry desquamat ion (G1)	HP
**43**	41	cT4N3M0 after operation pT2pN0M 0	Yes	2 Gy	50 Gy	No	Ery (G1)	HP
**44**	52	pT1cN0M0	No	2.5 Gy	42.5 Gy	9 Gy	Ery (G1)	Swelling
**45**	40	pT2N1M0	Yes	2 Gy	50Gy	10 Gy	Ery (G1)	HP
**46**	73	pT1cpN0M x	No	2.5 Gy	42.5 Gy	9 Gy	Ery (G1)	HP
**47**	58	pT1pN1	Yes	2.5 Gy	42.5 Gy	10 Gy	Ery (G1)	HP
**48**	57	pT DCIS N0M0	No	2.5 Gy	42.5 Gy	No	Ery (G1)	No reaction
**49**	44	pT2pN1M 0	No	2.5 Gy	30 Gy (disconti nued because of acute side effects)	No	Ery (G2), firing sensations,	No reaction
**50**	51	pT1b pN0 M0	No	2.5 Gy	42.5 Gy	No	G0	HP
**51**	63	pT2N0M0	Yes	2.5 Gy	42.5Gy	No	G0	HP, edema
**52**	75	pT1bpN0 Mx	No	2.7 Gy	40.5 Gy	No	G0	HP
**53**	56	pT1c pN0 pM0	No	2.5 Gy	42.5 Gy	10 Gy	Ery (G1)	No reaction
**54**	56	pT1b pN0	Yes	2.5 Gy	42.5 Gy	No	Ery (G1)	No reaction
**55**	61	pT1cN2 MX, luminal B	Yes	2 Gy	50 Gy	10 Gy	Ery (G1)	No reaction
**56**	55	pT2pN3a M0	Yes	2 Gy	50 Gy	10 Gy	G0	No reaction
**57**	72	pT1cN0M0	No	2 Gy	50 Gy	No	Ery (G2)	No reaction
