# Supplementary figures and images for: Assessment of Individual Radiosensitivity in Breast Cancer Patients Using a Combination of Biomolecular Markers
Source: Biomedicines. 2023 Apr 7;11(4):1122. doi: 10.3390/biomedicines11041122 (PMC10136353; doi:10.3390/biomedicines11041122)

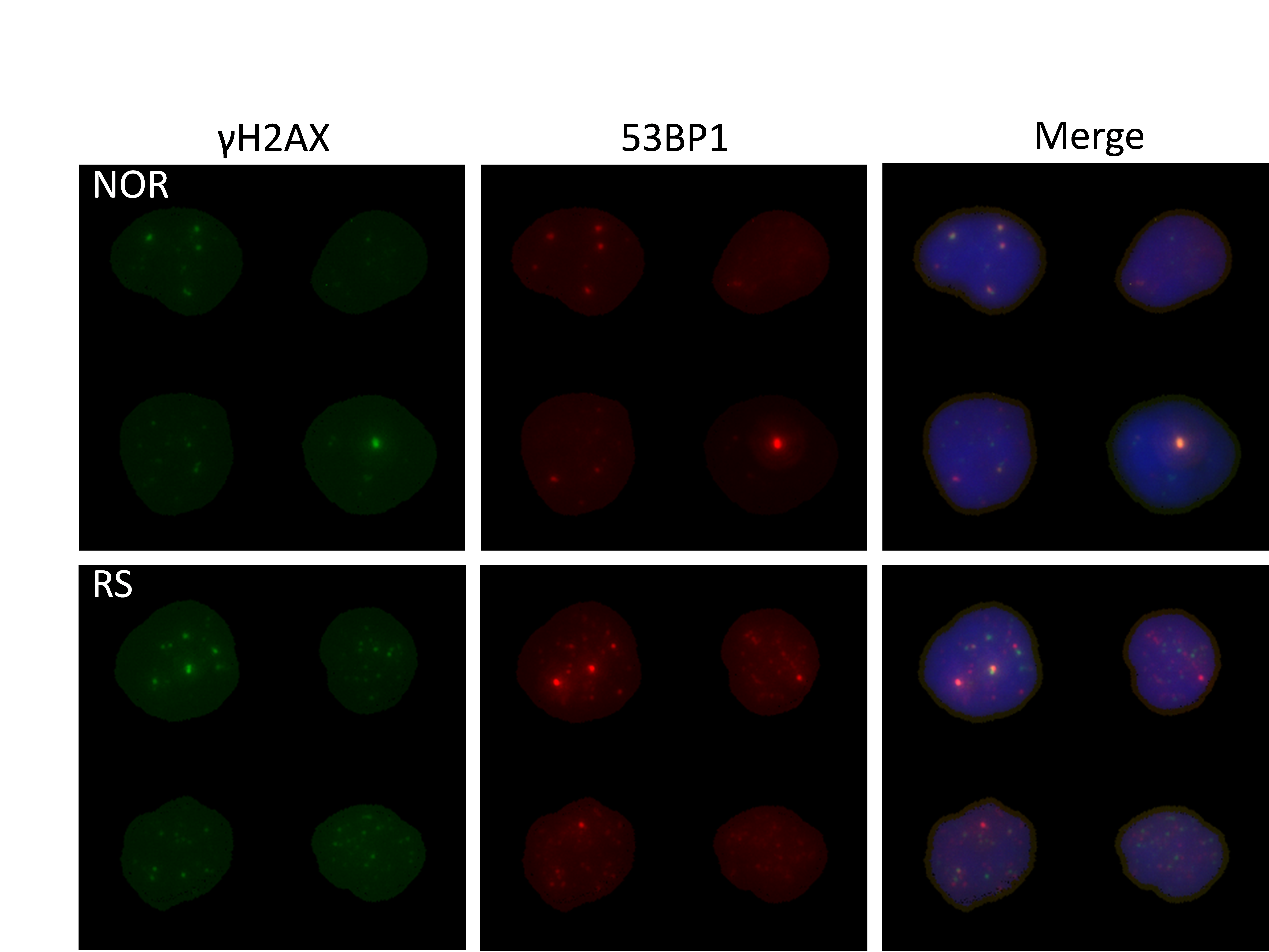

Supplement: Supplementary file 1 [file biomedicines-11-01122-s001.zip › Supplementary Figure S1 cell images Foci endogenous.png]

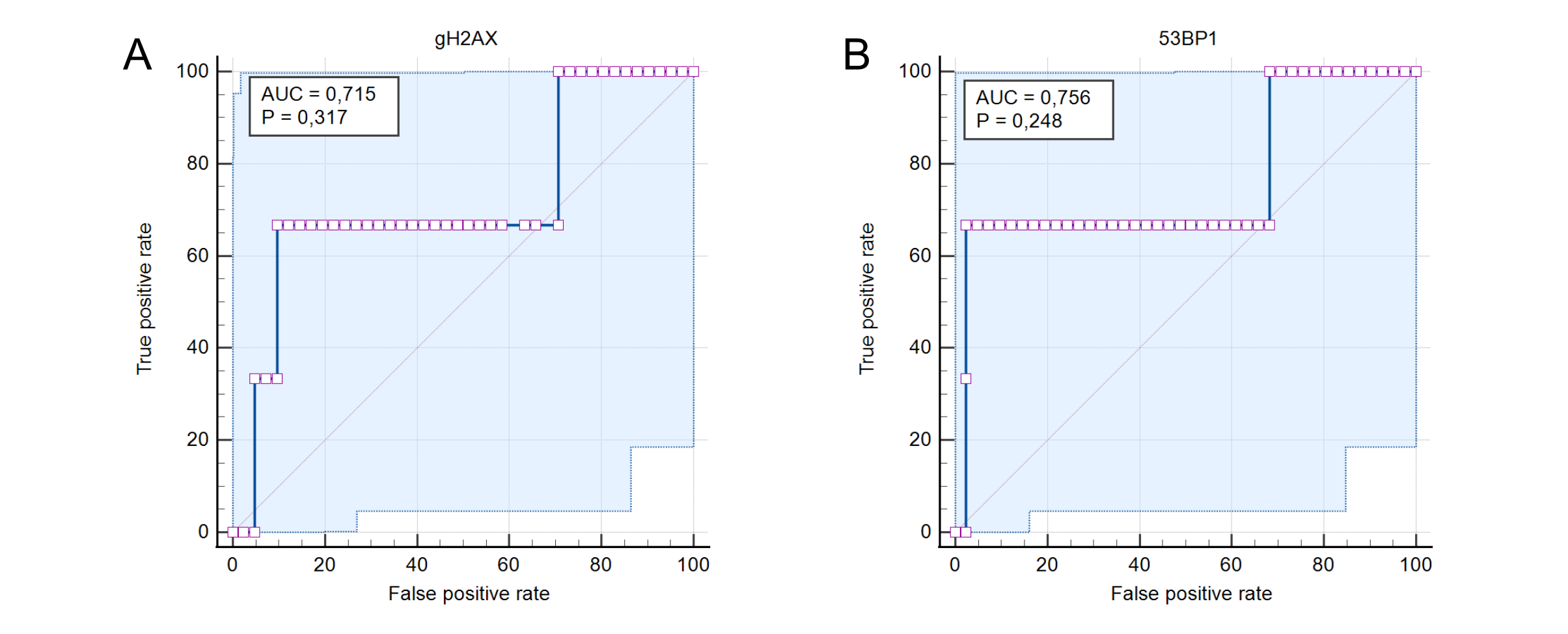

Supplement: Supplementary file 1 [file biomedicines-11-01122-s001.zip › Supplementary Figure S2 ROC analyses in vivo.png]

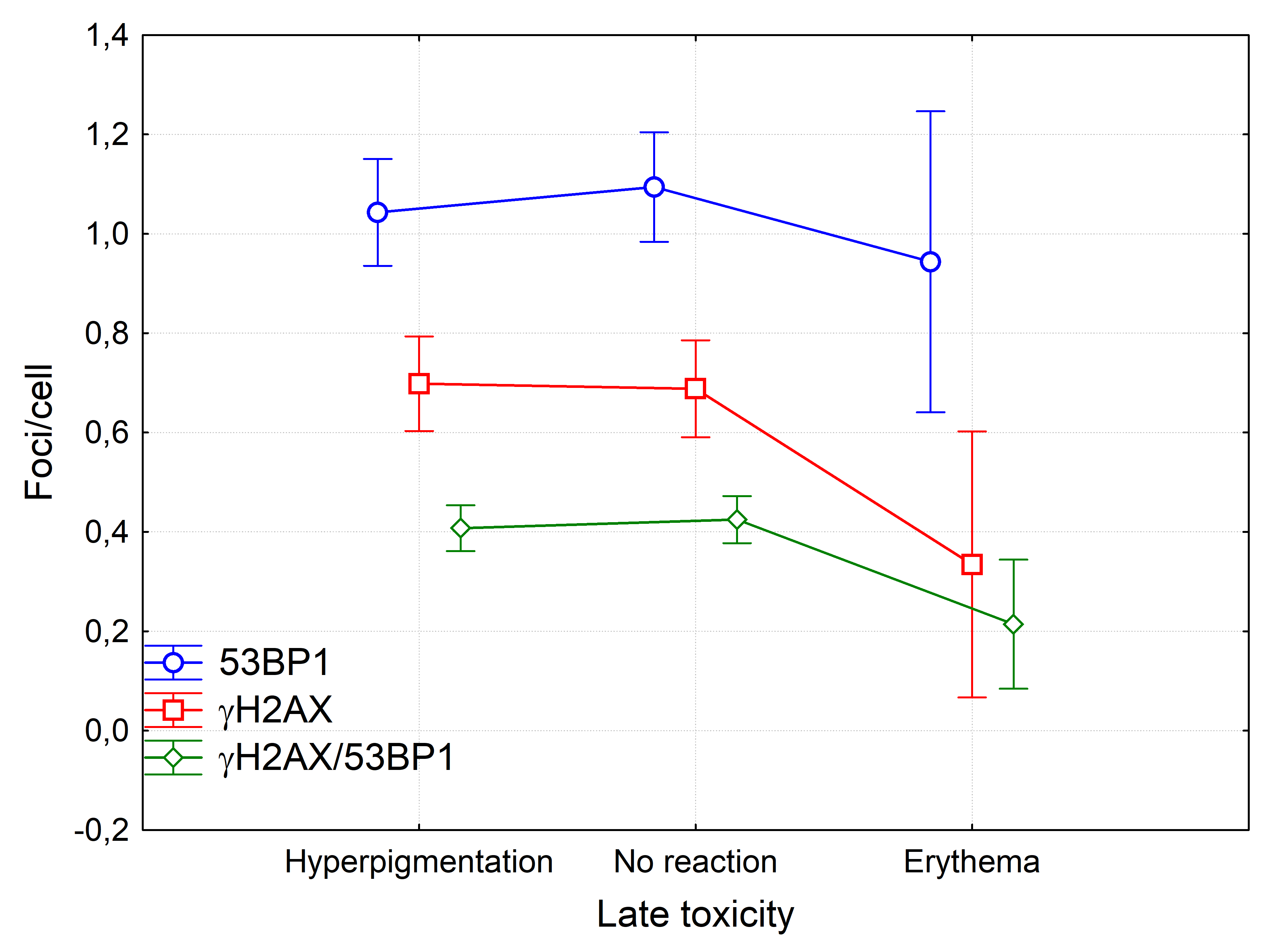

Supplement: Supplementary file 1 [file biomedicines-11-01122-s001.zip › Supplementary Figure S3 late toxicity.png]

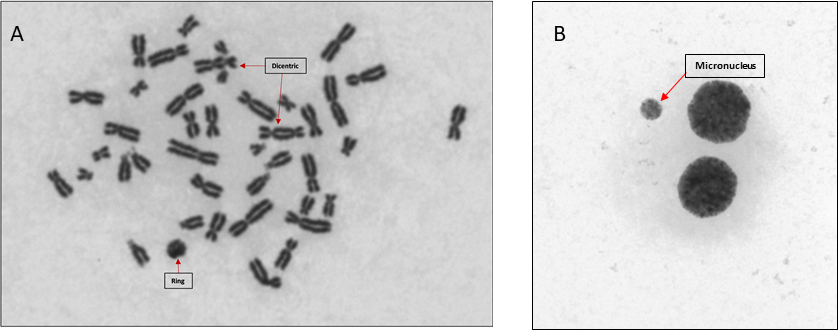

Supplement: Supplementary file 1 [file biomedicines-11-01122-s001.zip › Supplementary figure S4 Aberration pic cropped.png]

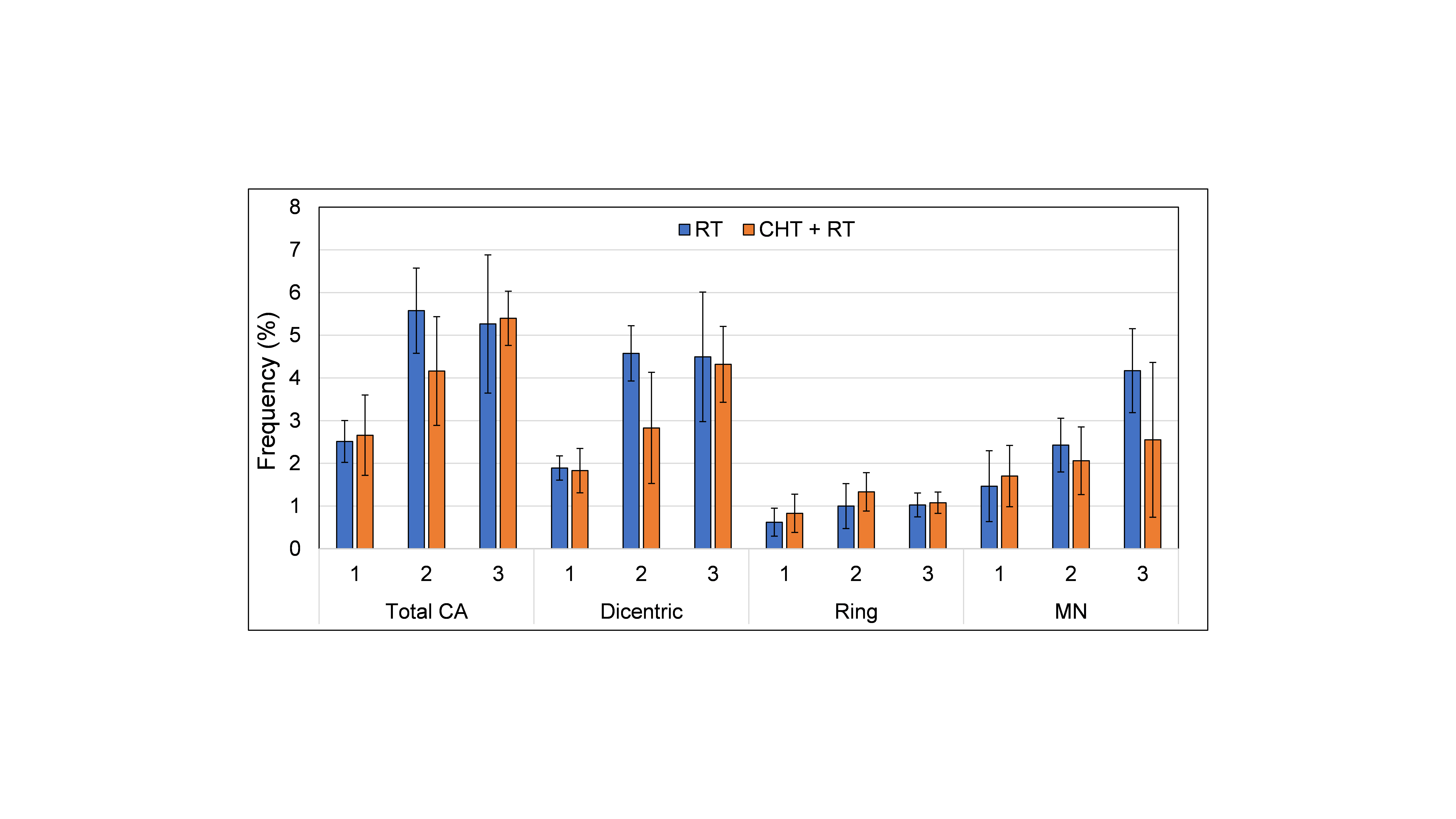

Supplement: Supplementary file 1 [file biomedicines-11-01122-s001.zip › Supplementary Figure S5 CA CHT+RT.png]

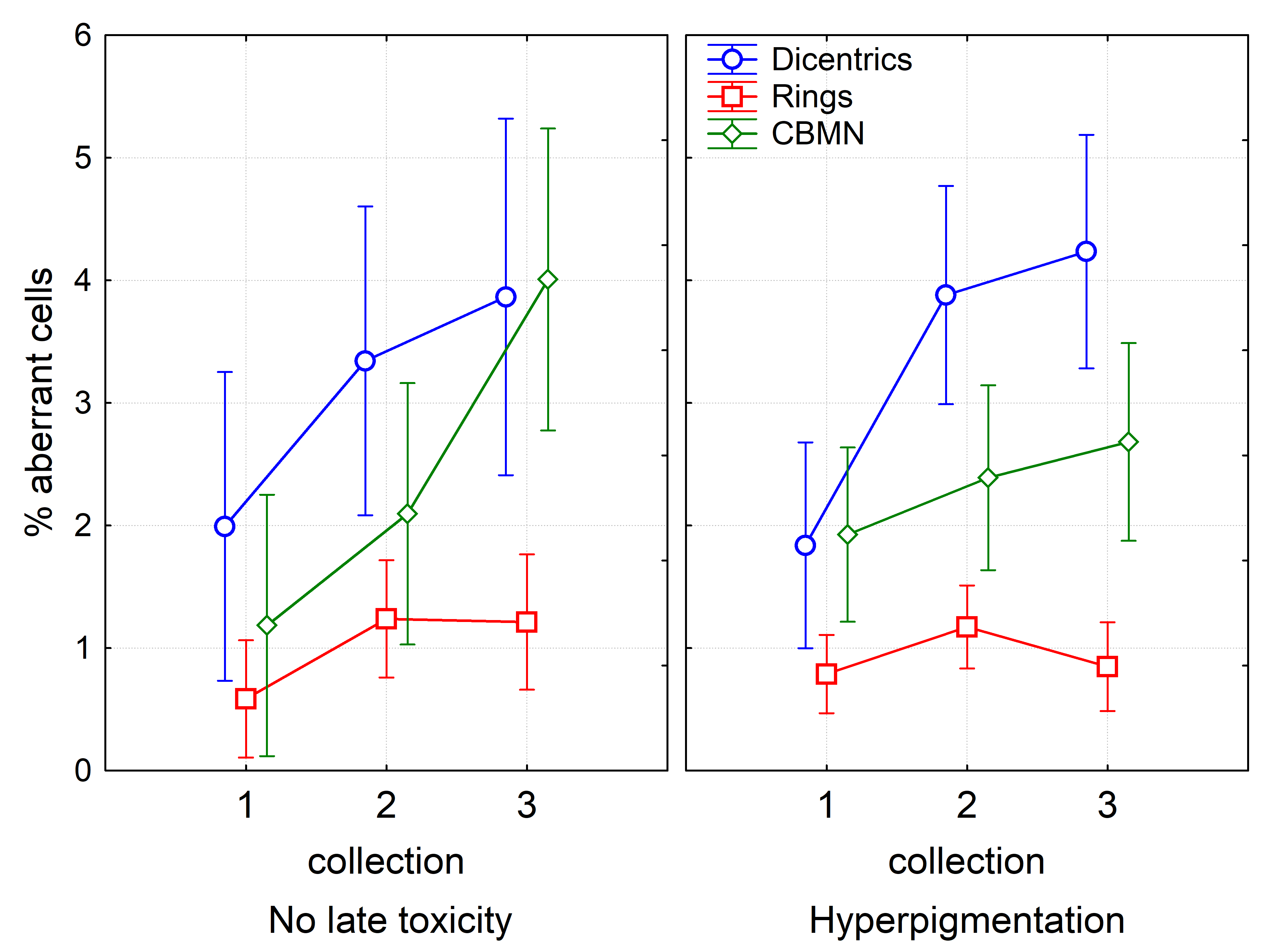

Supplement: Supplementary file 1 [file biomedicines-11-01122-s001.zip › Supplementary Figure S6 CA late toxicity.png]

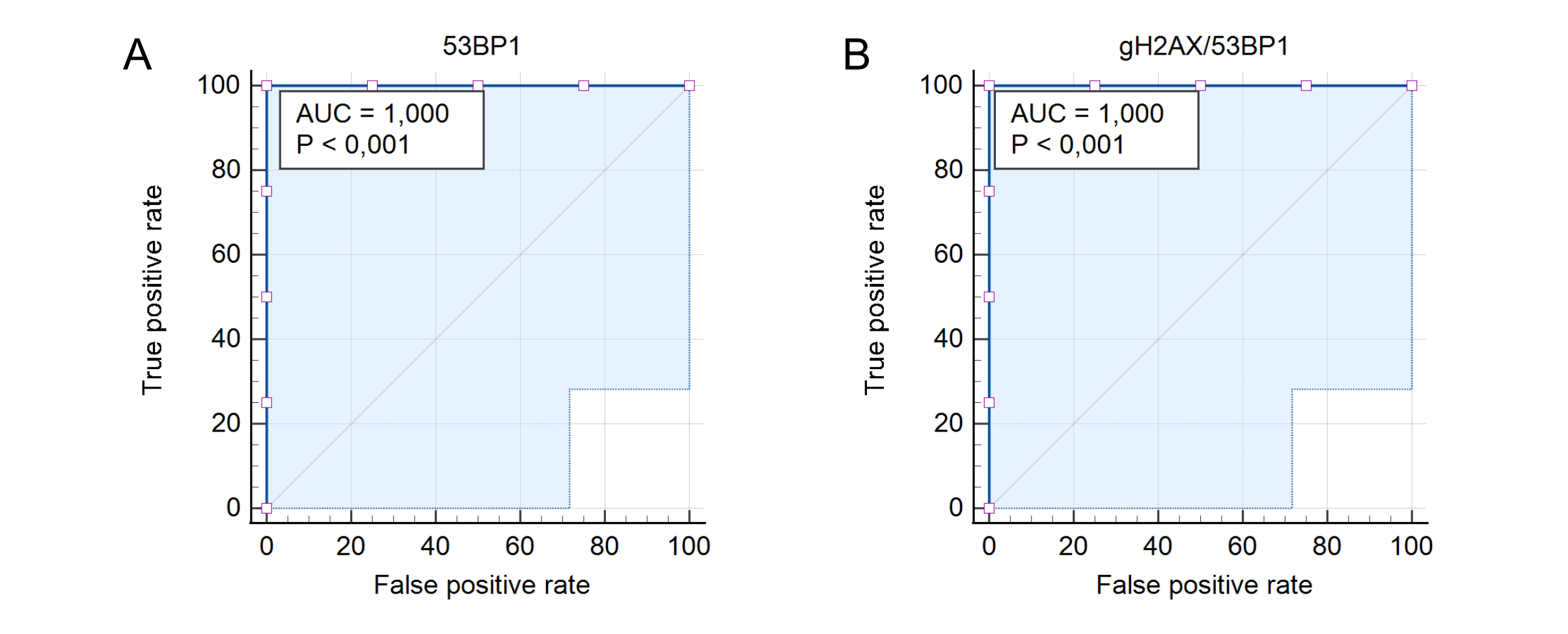

Supplement: Supplementary file 1 [file biomedicines-11-01122-s001.zip › Supplementary Figure S7 ROC analyses in vitro.png]

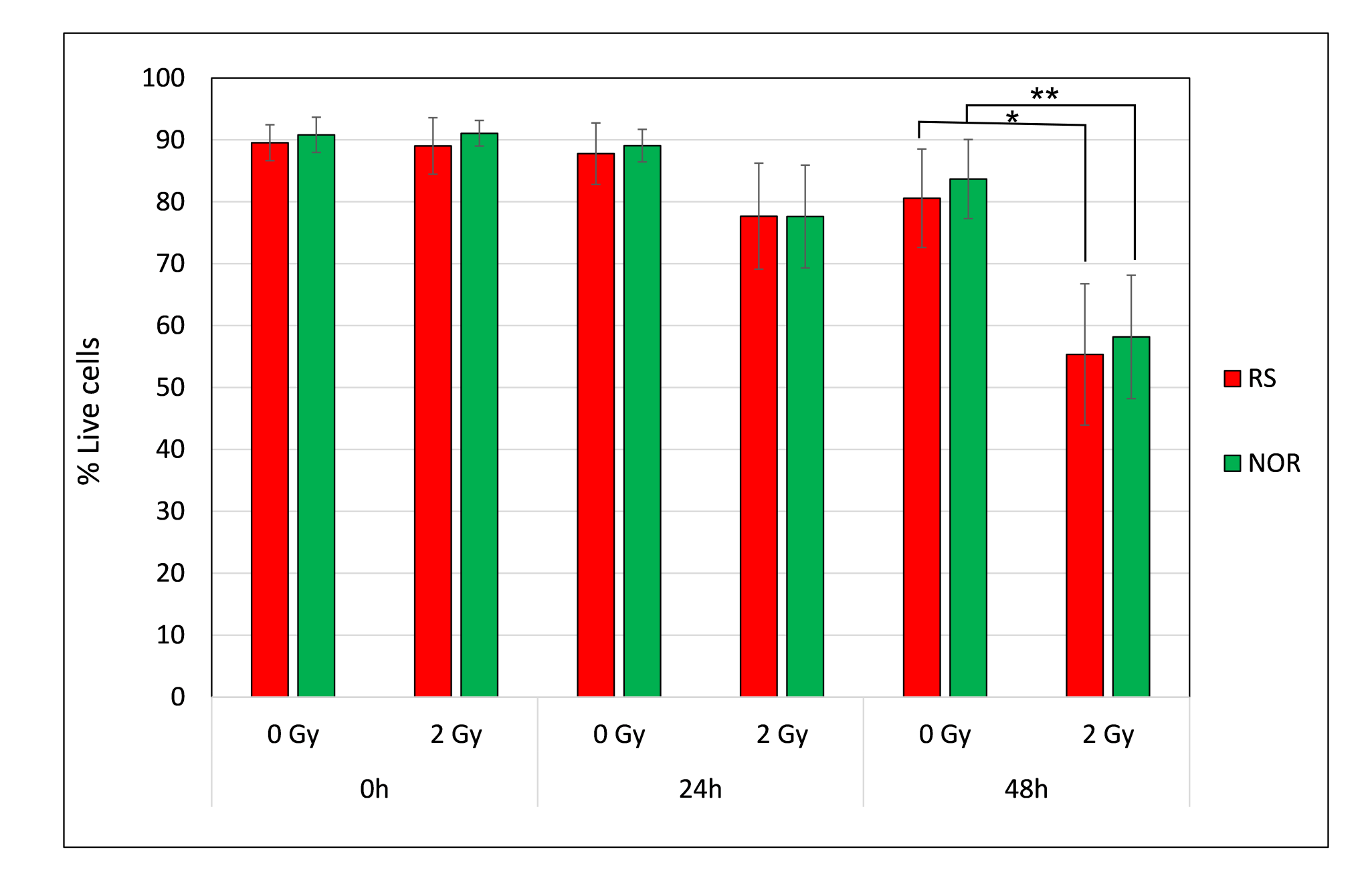

Supplement: Supplementary file 1 [file biomedicines-11-01122-s001.zip › Supplementary Figure S8 Apoptosis in vitro.png]
